# Psychobiological Correlates of Instructed Deceptive Responding: An Exploratory EEG Study

**DOI:** 10.3390/bs16081360

**Published:** 2026-08-09

**Authors:** Andrei Teodor Bratu, Florin Zamfirache, Nadina-Ionela Darie, Georgiana Dumitru, Gabriela Narcisa Prundaru, Cristina Dumitru, Beatrice Mihaela Radu

**Affiliations:** 1 Department of Anatomy, Animal Physiology and Biophysics, Faculty of Biology, University of Bucharest, Splaiul Independenţei 91-95, 050095 Bucharest, Romania; t.bratu20@s.bio.uibuc.ro (A.T.B.); zamfirache.florin@s.bio.unibuc.ro (F.Z.); prundaru.gabriela-narcisa@s.bio.unibuc.ro (G.N.P.); 2 Department of Psychology, Communication Sciences and Social Work, Faculty of Educational Sciences, Social Sciences and Psychology, The National University of Science and Technology POLITEHNICA Bucharest, Pitești University Centre, Targul din Vale, 1, 110040 Pitesti, Romania; nadina_ionela.darie@upb.ro; 3 Department for Teacher Training, The National University of Science and Technology POLITEHNICA Bucharest, Pitești University Centre, Targul din Vale, 1, 110040 Pitesti, Romania; georgiana.dumitru81@upb.ro; 4 Department of Educational Sciences, Faculty of Educational Sciences, Social Sciences and Psychology, The National University of Science and Technology POLITEHNICA Bucharest, Pitești University Centre, Targul din Vale, 1, 110040 Pitesti, Romania

**Keywords:** instructed deception, electroencephalography, frontal EEG, theta oscillations, trait anxiety, cognitive control

## Abstract

Intentional deceptive responding under laboratory instructions provides an experimental model for investigating the cognitive mechanisms involved in producing false responses, although it does not fully capture the complexity of real-world deception. Although deception has important implications for social functioning and has been widely studied, identifying reliable indicators that cannot easily be concealed remains a major challenge. In the present study, we investigated frontal EEG activity during intentional instructed deception and examined its association with stress-related and affective traits. The study included 86 healthy adults (M age = 26.97 years, SD = 9.42) who completed standardized self-report measures assessing depression (BDI), anxiety (BAI), perceived stress (PSS), and everyday deceptive behavior (LiES). A subsample of 48 participants subsequently completed a controlled truth–deception task while EEG activity was recorded from frontal scalp electrodes, providing an indirect measure of frontal electrophysiological activity. Absolute spectral power was analyzed across beta, alpha, theta, and delta frequency bands. The findings indicated that anxiety was positively associated with self-reported deceptive behavior, whereas depression and perceived stress were not significantly related to deception tendencies. EEG analyses suggested an increase in right-frontal scalp theta power during instructed deceptive responding, particularly through significant modulation of theta activity in the right hemisphere; however, later multilevel analysis showed it is a rather less specific activation appearing irrespective of the hemisphere. The increase remained significant when relative power was analyzed, indicating that the finding was robust to normalization of spectral power. Together, these findings suggest that instructed deceptive responding was associated with changes in frontal EEG oscillatory activity that are broadly consistent with mechanisms previously linked to executive control and conflict monitoring; however, because recordings were obtained using a low-density consumer EEG device during overt verbal responding, these interpretations should be considered exploratory. In this view, our study investigates habitual deceptive tendencies with affective traits and state-dependent EEG modulation during instructed deception, and our results could contribute to the understanding of emotion–cognition interactions in decisions involving uncertainty, social evaluation, and strategic information control.

## 1. Introduction

Deception constitutes a pervasive element of social interaction in human society, serving numerous roles that range from defense mechanisms and avoiding conflict to impression management and strategic cooperation between subjects. Although the ability to detect lies in an accurate manner has long attracted interest from a wide array of domains (e.g., scientific, legal, and clinical), reliably identifying deceptive behavior remains a considerable challenge stemming from the impossibility to directly and specifically identify physiological indicators of deception, relying instead on the use of indirect indicators that reflect broader emotional and cognitive processes (Vrij & Ganis, 2014). It is noteworthy that individual differences bring another dimension of difficulty for accurate evaluation, as gender differences, for instance, as highlighted by DePaulo et al. (1996), while not significantly impacting the frequency, can trigger distinct deceptive patterns depending on the purpose of the initiated deceptive behavior.

Research on deception has thus been developed along several unique methodological directions, each targeting different psychophysiological or behavioral by-products of deceptive behavior rather than the deception itself. Psychophysiological approaches have focused on orienting responses and autonomic indicators of arousal such as heart rate, skin conductance, and pupillary dilation, which tend to increase under uncertainty, perceptions of danger or evaluative processes (Duran et al., 2018). Behavioral frameworks have highlighted nonverbal cues associated with anxiety and cognitive processing as proper deception indicators, such as behavioral micro-expressions, gaze conduct, and postural state (Beattie, 2024; Ekman, 1988). Linguistic and discourse-analytic studies have examined changes in verbal flow and semantic complexity resulting from the increase in cognitive load and self-monitoring demanded by deceptive communication (DePaulo et al., 2003). At the same time, neurological approaches have aimed to characterize deception via the usage of functional neuroimaging and electrophysiological techniques by monitoring processes present in the neural systems supporting executive control, conflict monitoring, and response inhibition (Christ et al., 2009).

Despite significant empirical progress, none of these approaches have yet led to a reliable and context-generalizable indicator that can be used as certain evidence of deception in applied contexts. The main limitation is that many commonly monitored indicators—particularly aspects of autonomic arousal—are missing specificity, as they are significantly influenced by momentary stress, emotional volatility, and individual differences in anxiety processing, independent of the deceptive behavior per se (Kotsoglou & Biedermann, 2024). Recent reviews of neurophysiological deception research also emphasize that, although electroencephalography (EEG) and event-related potential markers manifest sensitivity to task manipulations in laboratory contexts, their diagnostic capacity remains limited by variability in task demands, motivational cues, and affective conditions (Taha et al., 2025).

Evidence from functional neuroimaging studies and meta-analyses suggests that deception is supported by a distributed neural network that includes prefrontal regions involved in executive control and inhibition, cingulo-opercular systems overseeing conflict monitoring, and limbic-memory structures such as the hippocampus and the amygdala that play an important part in affective evaluation (Christ et al., 2009; Meier et al., 2021; Tang et al., 2019). Within this relation, the prefrontal cortex (PFC) appears to play a leading role in suppressing prepotent truthful statements, maintaining alternative representations, and managing action selection under conditions of potentially highly socially impacting processes. Using EEG, activity in specific frequency bands has been linked to different functional operations relevant to deceptive behavior. Frontal theta oscillations have consistently been associated with cognitive control and conflict overseeing (Klimesch, 2012).

Importantly, deceptive behavior is tightly connected with the affective coordinates. Individual differences in anxiety processing, stress coping, and related personality traits can account for both the tendency to engage in deceptive behaviors and the neural resources demanded to sustain them. Recent evidence indicates that affective volatility and heightened perceived stress are associated with unique patterns of autonomic activation and frontal EEG asymmetry during tasks relating to deception and emotional processing (Bratu et al., 2025). Such findings relate to broader models of emotion regulation, which suggest that the PFC plays a key role in correlating cognitive control with the modulation of affective inputs under socially relevant conditions linked to performance or evaluation (Etkin et al., 2015; Shackman et al., 2011).

Taken together, these findings support the hypothesis that deceptive responding recruits executive-control processes involved in suppressing truthful responses. Because laboratory paradigms cannot reproduce the motivational, interpersonal and emotional complexity of real-world deception, the present study focuses specifically on instructed deceptive responding under controlled experimental conditions. Within this view, frontal EEG activity should be respondent not only to the cognitive demands of response creation and inhibition, but also to individual responses in psychobiological disposition. Accordingly, the present study investigates spectral changes in frontal EEG activity during intentional deception and examines their interaction with self-reported anxiety, perceived stress, and habitual deceptive behavior.

## 2. Materials and Methods

### 2.1. Objectives and Research Hypotheses

The present study aimed to characterize changes in frontal EEG activity correlated with deceptive behavior and to examine the psychophysiological factors that may interfere with these responses.

The current study was envisioned to address the following two primary objectives:

O1. To monitor the association between individual differences in psychobiological traits (anxiety, depression, and perceived stress) and the tendency to engage in everyday deceptive behavior.

O2. To investigate dynamics in frontal EEG activity across frequency bands during intentional deceptive behavior compared to truthful responses.

Based on the literature framework and the empirical objectives of the study, the subsequent hypotheses were formulated:

**H1.**  *Higher levels of anxiety will be positively associated with a greater propensity to engage in everyday deceptive behavior.*

**H2.**  *Depressive symptoms and perceived stress will not show significant associations with habitual deceptive behavior.*

**H3.**  *Female participants will report a higher propensity to engage in everyday deceptive behavior than male participants.*

**H4.**  *Intentional deception, compared to truthful responding, will be associated with increased frontal EEG activity, particularly in the right hemisphere.*

The proposed hypotheses were formulated a priori based on previous theoretical and empirical evidence rather than on the observed data. Hypotheses concerning anxiety, depression, stress, and frontal EEG activity were derived from studies implicating executive-control processes, affective regulation, and frontal oscillatory dynamics during instructed deceptive responding. The hypothesis regarding gender differences was included because the previous literature has reported sex-related differences in both emotional processing and deceptive behavior. In particular, women have been suggested to engage more frequently in prosocial deception aimed at maintaining interpersonal harmony, whereas men may more often employ self-serving deceptive strategies (DePaulo et al., 1996). Although these findings have not been entirely consistent across studies, they provided a sufficient theoretical rationale to examine potential gender differences in the present sample. Because previous findings regarding sex differences in deception remain mixed, this hypothesis was considered exploratory and intended to evaluate whether such patterns would also emerge within the present instructed-deception paradigm.

### 2.2. Participants

Eighty-six adult, healthy participants (mean age = 26.97, SD = 9.42; education, 26.7% high school, 32.6% associate degree, 34.9% master’s degree, 5.8% PHD; and gender, 77.9% female, n = 67) joined voluntarily in the study. None of the participants presented any history of mental conditions, either psychiatric or psychological. Informed consent was requested before participation, with the protocol being approved by the University of Bucharest’s Research Ethics Commission (Decision No. 85/10.11.2022). Of the total eighty-six participants, 48 provided recordings of sufficient quality for the follow-up EEG experiments, maintaining the overall structure of the entire sample for cross-comparisons to be possible in terms of age (mean age = 25.81, SD = 7.74), education (29.2% high school, 22.9% associate degree, 37.5% master’s degree, 10.4% PHD) and gender (79.2% female, n = 38). The participants were selected via nonprobabilistic, convenience methods, participating in the study on a voluntary basis after signing their respective agreements on participation and consent. In addition, they were cleared of any health status impeding the study such as heavy medication, neurological history, sleep disorders or substance abuse via the screening process.

To characterize the psychobiological profile of the sample and to evaluate the suitability of the data for subsequent parametric and correlational analyses, descriptive and distributional statistics were first examined for all demographic and questionnaire-based variables (Table 1). The full sample comprised 86 healthy adults with a mean age of 26.97 years (SD = 9.42), spanning a broad educational range from lower secondary to postgraduate level, thus providing adequate variability in sociodemographic background. From the total eighty-six participants, the 48 remaining participants included in the EEG phase maintained similar characteristics as a sample, allowing for cross-comparisons (mean age = 25.81, SD = 7.74).

As expected, participants reported subclinical levels of symptoms associated with depression (BDI: M = 13.43, SD = 9.62) and anxiety (BAI: M = 14.05, SD = 11.36), as well as having moderately perceived stress (PSS: M = 35.07, SD = 12.10). Self-reported day-to-day deceptive behavior, as targeted by the LiES, showed important interindividual variability (M = 64.80, SD = 28.03), indicating that the tendency to engage in deceptive behaviors in usual conditions was widely distributed across the sample rather than having a clear trend going into a certain direction. Additionally, the participants in the secondary subsample had a reasonable similarity with the overall sample, allowing for cross-analyses: BDI (M = 14.33, SD = 9.88), BAI (M = 14.60, SD = 12.38), PSS (M = 35.87, SD = 13.01), and LiES (M = 65.52, SD = 31.11) were all in line with the overall sample, thus allowing extensive further analyses.

Analysis of skewness and kurtosis values showed moderate changes from normality for several variables, most notably in the case of anxiety and age, which exhibited positive skewness and elevated kurtosis. These observations were confirmed afterwards by Shapiro–Wilk tests, which indicated significant deviations compared to a normal distribution for most measures (all *p* < 0.05), except for LiES scores, which resembled normality in a general sense. None of the distributions manifested extreme asymmetry or floor/ceiling effects, importantly, and observed minimum and maximum values confirmed a wide and continuous range of scores present for all the psychometric variables investigated.

The results of the statistical distribution highlight a heterogeneity of affective states and deceptive tendencies, while remaining within a regular range, specific to a non-clinical, natural sample. Some variables had statistical deviations from the norm, but the distribution, the effect size estimation and the rest of the analyses suggested the use of robust parametric procedures.

### 2.3. Instruments

Participants completed the following questionnaires prior to the experimental tasks:

Beck’s Depression Inventory (Beck et al., 2011) was used in order to assess the depression levels in a subclinical setting.

Beck’s Anxiety Inventory (Beck et al., 1988) was applied in order to evaluate the anxiety symptoms of the subject sample, especially relevant in the context of anxiety-induced artifacts during the testing sessions.

The Perceived Stress Scale (Cohen et al., 2014) was used to assess the present baseline stress level in the respondents, both in order to assure that the sample was not biased in this regard and to limit the possible impact on data analysis.

The Lying in Everyday Situations Scale (Hart et al., 2019) was applied to understand the initial propensity for engaging in deceptive behaviors as a control tool for their familiarity with deception in natural, social environments. The tests were applied online, via Google Forms, and the sections were thus counterbalanced in order to not induce any order effects.

### 2.4. Procedure

The experimental protocol included two successive components. First, participants completed an online battery of questionnaires assessing their psychobiological state and their self-reported propensity to engage in everyday deceptive behavior. Subsequently, a subsample of 48 clinically healthy individuals participated in an EEG experiment comprising two sessions based on an instructed truth–lie exercise inspired by the Control Question Test (CQT) paradigm (Raskin, 1978). This laboratory task, however, was not intended to reproduce forensic Control Question Testing, but rather to provide a standardized instructed-deception paradigm. During these sessions, participants responded to a series of ten simple, emotionally neutral questions (e.g., name, personal characteristics, preferences, and daily activities), answering truthfully in one condition and deceptively in the other with a maximum duration of 10 s for each response. The order of the truth and deception conditions was counterbalanced across participants to control for any order effects. Before the two sessions, participants completed a practice block of 5 distinct emotionally neutral questions alongside standardized instructions regarding answering as per the current stage the respondent was in, in order to familiarize the participants with the conversational style of the experiment following a truth–lie exercise inspired by the CQT paradigm, responding only with “Yes” or “No”. The same questions were again asked for the second run, which allowed a direct differentiation between truth and deceptive behaviors as a control measure. Each trial began with the presentation of a question addressed verbally, followed by the participant’s verbal response. Trials were separated by an inter-trial interval of 5 s. A one-hour interval separated the two sequential tests to minimize carry-over effects. Continuous EEG recordings were visually inspected, and recordings containing excessive movement or muscle artifacts were excluded from further analyses.

Electroencephalographic (EEG) signals were recorded using a Muse Headband 2 system (sampling rate: 256 Hz; InteraXon, Toronto, ON, Canada) with sensors positioned over frontal—AF7, AF8—and temporal scalp regions—TP9, TP10—and no mapping of the electrode placement was required due to the nature of the headset, which has a standard placement. The raw data were band-pass-filtered between 0.5 and 50 Hz and processed using artifact screening and segment-level quality control. Absolute spectral power was then computed for the delta, theta, alpha, and beta frequency bands by means of Fast Fourier Transform (FFT).

### 2.5. Statistical Procedures

After the data collection, the data underwent a quality-control process, and once the data passed it successfully, statistical procedures were applied using JASP software (version 0.98), encompassing procedures such as *t*-tests, descriptive statistics and inferential analysis. Although the Shapiro–Wilk test indicated departures from normality, the skewness and kurtosis values for the analyzed variables remained within acceptable limits (±3), suggesting that deviations from normality were not severe. Given the relatively balanced sample size and the well-established robustness of parametric procedures to moderate violations of the normality assumption, Pearson correlations and independent/dependent Student’s *t*-tests were retained for the analyses. Previous methodological research has shown that parametric tests generally maintain appropriate Type I error rates and statistical power under such conditions, particularly when skewness and kurtosis remain within acceptable ranges.

For the EEG data, spectral power was computed using a classical Fast Fourier Transform applied to the complete artifact-cleaned signal segment for each condition and electrode. No additional epoching procedure was applied before FFT computation; therefore, the analyzed segment corresponded to the full available recording for each truth and instructed-deception condition. Because the FFT was applied directly to the full signal segment, no overlapping windows were used, and no window-based epoch rejection was performed. Artifact control was performed before spectral estimation, at the recording/segment level, by excluding recordings with insufficient signal quality, unstable electrode contact, excessive movement contamination or visually evident non-physiological noise. Of the 86 recruited participants, 21 were excluded because of excessive movement artifacts, 9 because of unstable electrode contact, 5 because recordings were incomplete, and 3 because of technical failures. This procedure resulted in a final EEG sample of 48 participants. No tapering window (e.g., Hann or Hamming) was applied before FFT because spectral estimation was performed on the complete cleaned signal segment. Spectral estimates were obtained from continuous recordings separately for the truth and instructed-deception conditions. EEG data were analyzed during overt verbal responding. Because spectral analysis was performed on continuous recordings without epoching, recording quality was evaluated at the whole-recording level. Only recordings maintaining stable electrode contact and acceptable signal quality throughout the experiment were retained for analysis. The quality-control procedure was completed before spectral analysis and independently of the experimental outcomes, thereby minimizing the risk of selection bias.

The Muse headset has been previously validated against research-grade EEG systems and has demonstrated adequate reliability and sensitivity for the assessment of event-related and oscillatory brain activity in both cognitive and affective tasks (e.g., Krigolson et al., 2017, 2021). Absolute power was also used in order to monitor the amplitude of electrophysiological oscillations within specific frequency bands, which was advantageous when working with consumer-grade EEG devices, which have limited electrode coverage and may not fully support reliable referencing across multiple cortical regions. Additionally, because Muse 2 recordings can be sensitive to signal quality issues, absolute power provides a more stable and reproducible measure for within-subject analyses; thus, it was preferred because it may alleviate some of the limitations offered by Muse 2 usage.

Before the inferential testing, all variables were inspected for any missing values, outliers, and distributional properties. Shapiro–Wilk tests were used in order to assess the normality of the psychometric and EEG variables, as well as skewness and kurtosis indices. Given the partial deviations from normality, both parametric robustness and effect sizes were considered in the interpretation of results.

Descriptive statistics (skewness, kurtosis, means, standard deviations, minimal and maximal values) were computed for all demographic, psychometric, and electrophysiological variables. Associations between psychobiological traits (BDI, BAI, PSS) and everyday deceptive behavior (LiES) were examined using Pearson correlation coefficients, with 95% confidence intervals and Fisher’s z-transformed effect sizes. Gender differences in psychometric measures and LiES scores were tested using independent-samples *t*-tests. Homogeneity of variance was assessed using the Brown–Forsythe test, and adjusted degrees of freedom were reported when required. Effect sizes were presented as Cohen’s d. For the EEG analyses, absolute spectral power was calculated for the alpha, delta, beta, and theta frequency bands at left and right frontal sites under both truthful and deceptive conditions. Within-subject comparisons between conditions were assessed using paired-samples *t*-tests for each frequency band and hemisphere. Additional paired *t*-tests were performed to evaluate hemispheric differences (left vs. right) separately for the deceptive and truth conditions. Effect sizes for within-subject differences were computed as Cohen’s d for dependent samples.

All statistical tests were two-tailed, with the significance threshold set at *p* < 0.05. Where necessary, effect sizes and confidence intervals were reported to facilitate interpretation of the size and reliability of measured effects.

To control for multiple comparisons, false discovery rate (FDR) corrections were applied separately to families of tests (EEG paired comparisons and psychometric correlations). To control the false discovery rate arising from multiple statistical tests, Benjamini–Hochberg FDR correction was applied separately within each predefined family of analyses. One family comprised the six psychometric correlation analyses, whereas a second family comprised the eight paired EEG comparisons between truthful and deceptive responding (four frequency bands × two hemispheres). Raw *p*-values and FDR-adjusted *p*-values are both reported in the Results tables.

To examine the relationship between electrophysiological responses and individual differences, difference scores (deception vs. truth) were computed for the different bands. These indices were then correlated with psychometric measures.

To assess robustness to deviations from normality, nonparametric Wilcoxon signed-rank tests were conducted as sensitivity analyses for the key EEG comparisons.

## 3. Results

### 3.1. Associations Between Everyday Deceptive Behavior and Psychobiological Traits

**Figure 1:**
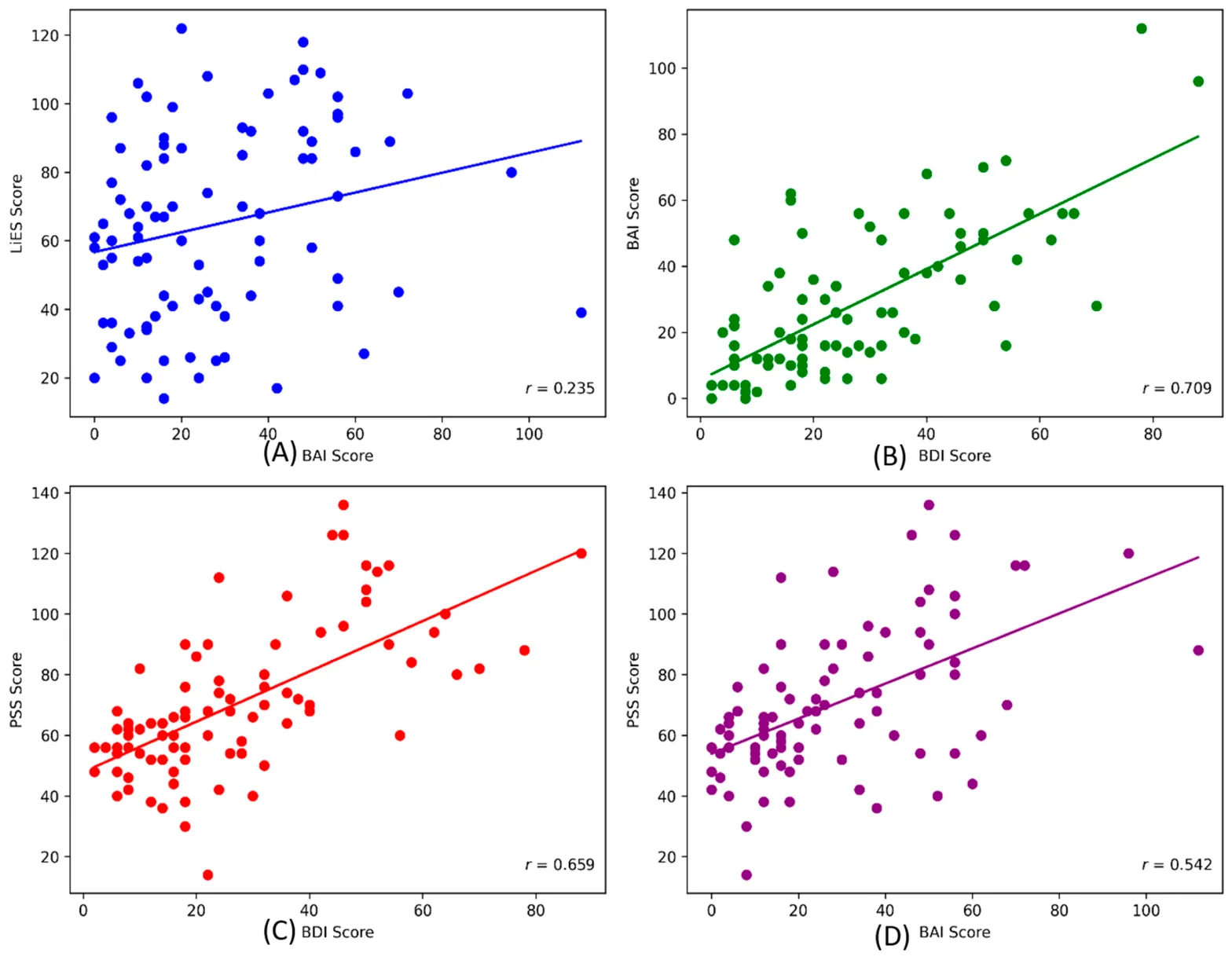
Correlations between affective traits and deceptive behavior. Scatter plots illustrate relationships between psychometric measures: (**A**) anxiety (BAI) and deceptive behavior, (**B**) depressive symptoms (BDI) and anxiety (BAI), (**C**) depressive symptoms (BDI) and perceived stress (PSS), and (**D**) anxiety (BAI) and perceived stress (PSS). Lines represent regression fits; r indicates Pearson correlation coefficients.

We analyzed the correlation levels of depressive symptoms, anxiety symptoms, perceived stress, and LiES scores of the entire sample (N = 86) to assess individual affective differences and engagement in daily deceptive behavior. The results presented in Figure 1 and Table 2 show positive relationships between depression, anxiety, and perceived stress, as expected, explained by their common comorbidity. Depressive symptoms correlated significantly with anxiety (r = 0.709, *p* < 0.001) and perceived stress (r = 0.659, *p* < 0.001), and anxiety correlated significantly with perceived stress (r = 0.542, *p* < 0.001).

The model was more selective for habitual deceptive behavior, thus indicating the differential association of different affective dimensions with deceptive tendencies. Anxiety had a lower association with LiES scores (r = 0.235, *p* = 0.030) but was statistically significant, indicating that individuals with significant anxiety scores had a higher tendency towards everyday deceptive behaviors. The association between anxiety and everyday deceptive behavior remained significant after FDR correction, *r* = 0.235, *p* = 0.030, *p* FDR = 0.045. Additionally, Spearman correlation was additionally conducted, reflecting similar patterns (Spearman’s rho = 0.27, *p* = 0.01). Spearman’s correlation subsequently uncovered a relation between deceptive behavior and depression, with a 0.32 Spearman’s rho and *p* = 0.02.

Depression and stress had no significant correlation with self-reported deceptive behavior (BDI: r = 0.134, *p* = 0.220; PSS: r = 0.073, *p* = 0.505). Negative emotionality and subjective stress level did not correlate with habitual deception, the confidence interval was not significant and the Fisher z effect size was also small.

### 3.2. Gender Differences in Psychobiological Traits and Deceptive Behavior

To explore potential gender-related discrepancies in affective traits and in the tendency to engage in day-to-day deception, independent-samples *t*-tests were used for depressive symptoms, anxiety, perceived stress, and LiES scores (Table 3). This analysis had the objective to assess whether previously reported sex-related variations in emotional reactivity and social conduct were also reflected in self-reported deceptive tendencies within the current sample.

A significant gender difference emerged for anxiety, with female participants reporting higher BAI scores than males, t(84) = 2.271, *p* = 0.026, reflected by a medium effect size (Cohen’s d = 0.590). The Brown–Forsythe assessment indicated a violation of the homogeneity of variance assumption for this analysis, and adjusted statistics were therefore taken into consideration, confirming the robustness of the effect. No significant gender differences were present for depressive symptoms or perceived stress, although both measures showed numerically higher trends in females.

In contrast to these affective differences, no significant gender-related variation was found in everyday deceptive behavior. LiES scores did not differ depending on the gender of the participants, t(84) = −0.55, *p* = 0.583, indicating comparable self-reported frequencies of deception across genders.

### 3.3. Frontal EEG Spectral Dynamics During Truthful and Deceptive Responding

Absolute spectral power analysis measured with EEG in the frontal regions was used to investigate the correlations between electrophysiological measures and deception. Measures of all frequency bands (delta, theta, alpha and beta) of both hemispheres were tracked in both deceptive and truth conditions.

The absolute mean values of spectral power recorded in the truth condition ranged from 69.21 (SD = 18.81) to 81.29 (SD = 17.52) for the left hemisphere and from 69.65 (SD = 15.74) to 82.27 (SD = 12.65) in the right hemisphere in the first recording for the truth condition. The highest values were recorded in the delta and beta bands. Alpha and beta values recorded at the left hemisphere level showed moderate deviations (skewness = −1.70, kurtosis = 8.48; W = 0.833, *p* < 0.001 for alpha and skewness = −1.02, kurtosis = 3.64; W = 0.923, *p* = 0.004 for beta), although the distributions were valid for intra-subject comparisons.

For the deception data, overall absolute spectral power values were higher across most frequency bands. Right-hemispheric theta power increased from M = 71.88 (SD = 12.47) in the truth condition to M = 76.15 (SD = 13.03) during deception, while right-hemispheric alpha absolute spectral power rose from M = 78.20 (SD = 11.33) to M = 80.71 (SD = 9.65). Left-hemispheric theta absolute spectral power showed a smaller increase (from M = 73.22, SD = 13.11 to M = 74.86, SD = 15.73), and left alpha increased from M = 77.96 (SD = 13.13) to M = 80.37 (SD = 13.10). The distribution values for the measurements in the deceptive condition were closer to normal values. The Shapiro–Wilk values were insignificant (right theta: W = 0.963, *p* = 0.137; left beta: W = 0.968, *p* = 0.210).

A unified overview of central tendency and variability across both conditions (Table 4) indicated comparable interindividual dispersion, with similar coefficients of variation in the truth and deception tasks (e.g., right theta: CV = 0.174 vs. 0.171; right alpha: CV = 0.145 vs. 0.120). The largest mean increases from Condition 1 to Condition 2 were observed in the right-hemispheric theta band (ΔM = +4.27) and right-hemispheric alpha band (ΔM = +2.51).

**Figure 2:**
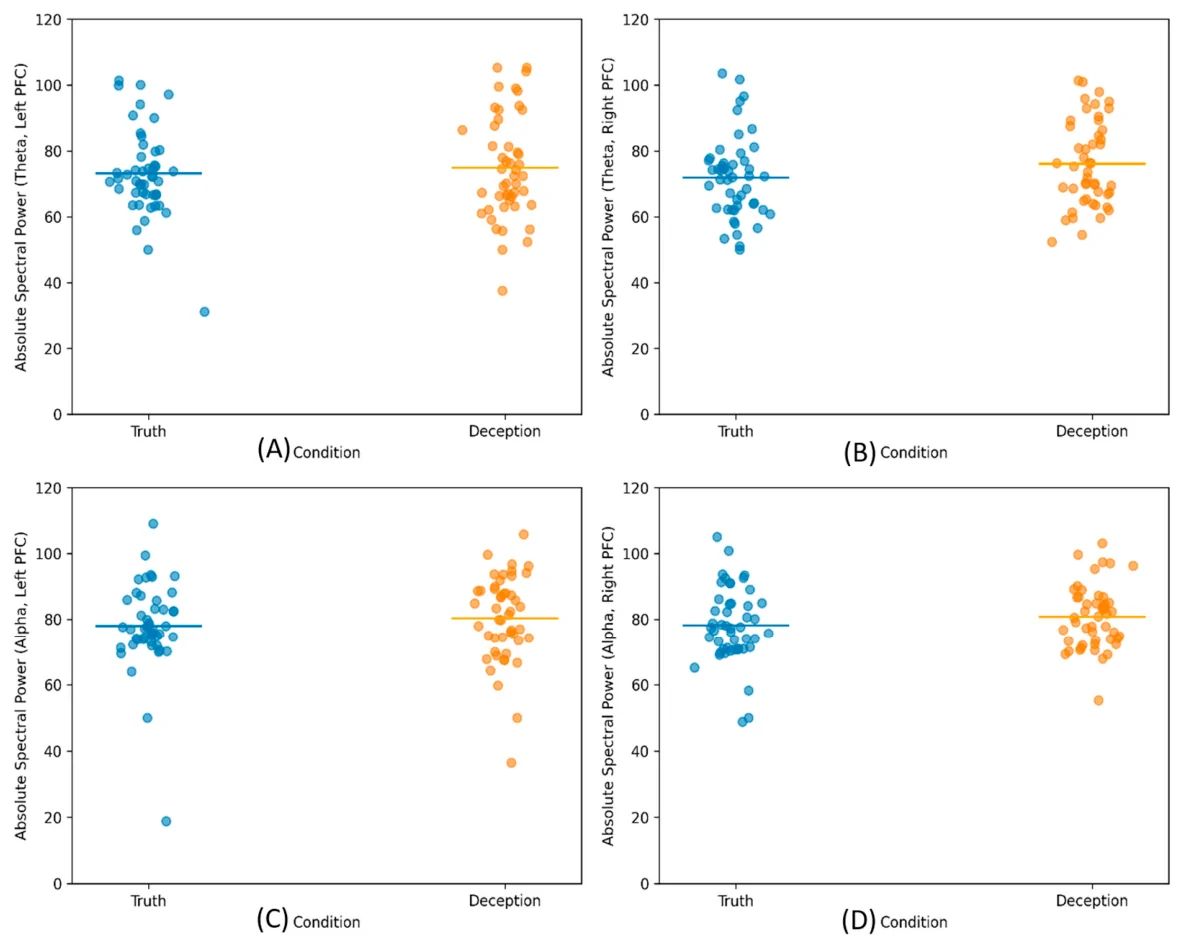
Frontal absolute spectral power (μV^2^) during truthful and deceptive responding. Individual and mean values of absolute spectral power in the alpha and theta frequency bands recorded over the left and right frontal sites during truthful (Truth) and deceptive (Deception) responding (N = 48). Each dot represents an individual participant; horizontal lines indicate condition means (Deception shown in orange). Panels display (**A**) left-frontal theta, (**B**) right-frontal theta, (**C**) left-frontal alpha, and (**D**) right-frontal alpha power.

Paired-samples *t*-tests (Table 5) confirmed that these descriptive increases reached statistical significance only in the right hemisphere. As illustrated in Figure 2, deceptive responding was associated with significantly higher right-frontal absolute theta power, t(47) = −3.02, *p* = 0.004, Cohen’s d = −0.436, and higher right-frontal absolute alpha power, t(47) = −2.02, *p* = 0.049, d = −0.292; however, the alpha effect did not survive FDR correction, with a recalculated *p* FDR = 0.245. Importantly, the right-frontal theta increase remained significant in a nonparametric Wilcoxon signed-rank sensitivity analysis, *p* = 0.019. No significant condition-related differences were observed for left-hemispheric theta (t(47) = −0.93, *p* = 0.357) or alpha activity (t(47) = −1.78, *p* = 0.081), nor for beta bands in either hemisphere (all *p* > 0.10). The pattern depicted in Figure 2 highlights frequency- and hemisphere-dependent modulation of frontal oscillatory activity during intentional deception.

Additional analyses comparing left and right frontal areas were performed for both the truth and lie conditions to assess differences in effects for the respective hemispheres and modulations specific to each condition (Table 6). In the case of deception, no significant differences were detected for any frequency band, indicating that the absolute asymmetries measured under the lie condition did not reach a statistically significant level. However, absolute spectral power increased more than in the truth condition.

A similar result was observed for the measurements for the truth condition (Table 7). Pairwise comparisons showed no significant differences between EEG recordings of different frequency bands (e.g., theta: t(47) = −0.64, *p* = 0.523; alpha: t(47) = −0.11, *p* = 0.916).

As a sensitivity analysis, the primary comparison of right-frontal theta activity was repeated using relative theta power. Relative theta power was significantly higher during deceptive responding (M = 0.194, SD = 0.018) than during truthful responding (M = 0.188, SD = 0.018). A paired-samples *t*-test confirmed that this increase remained statistically significant, t(47) = −2.76, *p* = 9.008, Cohen’s d = 0.40, indicating that the main finding was not dependent on the use of absolute spectral power, as seen in Table 8.

### 3.4. Order Effects

To examine whether task order influenced the observed EEG effects, a mixed repeated-measures ANOVA was conducted with Condition (truth vs. deception) as the within-subject factor and Order (Truth–Deception vs. Deception–Truth) as the between-subject factor. A significant main effect of Condition was observed, F(1, 46) = 8.79, *p* = 0.005, confirming higher right-frontal theta power during deception. Neither the main effect of Order, F(1, 46) = 2.08, *p* = 0.156, nor the Condition × Order interaction, F(1, 46) = 2.08, *p* = 0.857, reached statistical significance, indicating that the effect of deception was independent of task order.

### 3.5. Multilevel Analysis

To further investigate whether deception-related EEG activity differed as a function of hemisphere and frequency band, as seen in Table 9, a 2 (Condition: truthful vs. deceptive) × 2 (Hemisphere: left vs. right) × 4 (Band: delta, theta, alpha, beta) repeated-measures ANOVA was conducted. Because Mauchly’s test indicated violations of sphericity for effects involving Band, Greenhouse–Geisser corrections were applied where appropriate. The analysis revealed a significant main effect of Condition, F(1, 47) = 5.878, *p* = 0.019, ηp^2^ = 0.111, and a significant main effect of Band, F(1.404, 66.007) = 14.718, *p* < 0.001, ηp^2^ = 0.238. No significant main effect of Hemisphere was observed, F(1, 47) = 0.007, *p* = 0.935. Furthermore, no significant interactions were found between Condition and Hemisphere, F(1, 47) = 0.078, *p* = 0.781, Condition and Band, F(2.539, 119.321) = 0.878, *p* = 0.440, Hemisphere and Band, F(2.026, 95.217) = 0.549, *p* = 0.582, or the three-way interaction, F(2.440, 114.692) = 1.978, *p* = 0.133.

Therefore, the significant paired comparison observed for right-frontal theta should be interpreted as a focused post hoc finding rather than evidence for a significant global Condition × Hemisphere × Band interaction.

### 3.6. Regression Analysis Across Indicators

To determine whether anxiety predicted deceptive tendency independently of depressive symptoms and perceived stress, a multiple linear regression was performed with LiES as the dependent variable and BAI, BDI, and stress as predictors. The overall model was not significant, F(3, 44) = 1.818, *p* = 0.158, explaining 11.0% of the variance (R^2^ = 0.110). None of the predictors reached statistical significance (Table 10).

Thus, the association between anxiety and deceptive tendency was not maintained after simultaneously accounting for depressive symptoms and perceived stress, indicating that the observed bivariate relationships should be interpreted with caution.

A multiple linear regression analysis was conducted to examine whether anxiety (BAI), depression (BDI), perceived stress (PSS), and the propensity to deceive (LiES) predicted relative theta power. The overall regression model was not statistically significant, F(4, 43) = 0.696, *p* = 0.599, explaining only 6.1% of the variance in relative theta power (R^2^ = 0.061, adjusted R^2^ = −0.027). None of the individual predictors reached statistical significance, including anxiety (β = 0.137, t = 0.494, *p* = 0.624), depression (β = 0.166, t = 1.059, *p* = 0.295), perceived stress (β = −0.174, t = −0.870, *p* = 0.389), or LiES scores (β = −0.144, t = −0.590, *p* = 0.558), as noted in Table 11.

## 4. Discussion

The study investigated the relationships between psychobiological and electrophysiological measurements for frontal scalp EEG activity during the intentional deception test. Both individual differences in affective traits and the dynamics of EEG values recorded in different truth and lie conditions were measured. Two main results were highlighted from the analyses, one of which was that anxiety was positively associated with self-reported habitual deceptive behavior. The second observation indicates the occurrence of higher right-frontal scalp theta activity, which the multivariate analyses later concluded is a rather less specific overall activation. These effects were observed specifically under the instructed-deception condition of the present paradigm. Although depression, anxiety, and perceived stress are interconnected and often occur together, only anxiety is directly linked to self-reported deceptive behaviors; however, due to the lack of statistical significance in the multiple regression analysis, anxiety cannot be considered a specific or selective correlate of deception.

The results of the correlation between habitual deception and anxiety could suggest that instructed deceptive behavior may be associated with affective–motivational regulation rather than the manifestation of a general indicator of psychologically stressful conditions; however, due to the lack of impact of anxiety as a predictor it is hard to establish causality; thus, Hypothesis 1 (H1) is only partially supported, which indicates the possibility that threat sensitivity and anticipatory vigilance may be related to reliance on information control schemes in enhanced social contexts.

Anxiety is associated with potential threats and increased monitoring of the environment and social cues, which may lead to the emergence of anticipatory mechanisms with a role in control and adaptation to the environment (Bishop, 2009; Shackman et al., 2011). The use of deception as a strategy for self-protection or image management in social situations thus appears as a strategy for adapting to situations perceived as risky or when the perceived cost of negative evaluation, punishment or status danger is negatively assessed. From an evolutionary point of view, anxiety increases vigilance and facilitates adaptive control in uncertain or novel environments. Thus, the ability to hide, alter, or strategically reveal information reduces risk and increases chances in risky social situations, which over time leads to the use of deception as a learned behavior (Nesse, 2019). Deception is closely related to processes of threat regulation and monitoring; it is not a mechanism triggered only by emotional situations. The lack of associations with situations such as stress or depression hints towards the specificity of this mechanism. These results partially support Hypothesis 2 (H2); however, the specificity of affective indicators involved in deceptive behavior has yet to be definitively confirmed in this study.

Although anxiety was associated with self-reported deceptive behavior, it did not significantly predict EEG modulation during deception, which may suggest a dissociation between dispositional tendencies and state-dependent electrophysiological responses.

Correlational analyses between EEG difference scores and psychometric measures were additionally performed and did not reveal significant associations (all *p* > 0.05), suggesting that individual differences in anxiety, perceived stress, deception, and habitual deception were not directly related to the magnitude of frontal oscillatory changes during deceptive responding.

At the frontal EEG activity level, the strongest effect observed during instructed deceptive responding came in the form of enhanced right-frontal theta power. Frontal oscillations have been linked to cognitive control, conflict monitoring, and the processing of top-down regulation, particularly when instinctive responses must be suppressed, which is somewhat similar to the current situation (Cavanagh & Frank, 2014). Within the present task, truthful responding may have represented an instinctive, dominant activated tendency, whereas deception may rely on the active inhibition of said tendency and the development of an alternative response. In the literature, the observed increased activity of the right-frontal theta band during deceptive responding reflects the increased demands on processes commonly associated with executive control involved in response monitoring, conflict processing, and goal-directed behavior under conditions of internal inconsistencies (Langleben et al., 2005; Meier et al., 2021). Although paired comparisons identified a significant right-frontal theta increase, the repeated-measures ANOVA did not reveal a significant Condition × Hemisphere interaction. Therefore, the apparent hemispheric specificity should be interpreted cautiously and requires replication using higher-density EEG recordings.

The significant frontal oscillatory effects are coherent with evidence that frontal activity plays a role in emotional regulation and control in the inhibitory aspects, especially in conditions where conflict, uncertainty, and potentially socially taxing situations can appear and can prove themselves harmful for the individual (Aron et al., 2014; Etkin et al., 2015). These processes could thus be less impacted by individual differences such as gender, as this study did not find a noticeable impact of gender on deceptive tendencies, with the results therefore not supporting Hypothesis 3 (H3), which predicted a higher propensity for everyday deception in female participants. The results indicate that although anxiety levels were higher in female participants, they were not more likely to engage in deceptive behaviors, so gender effects alone are not a determinant of habitual deception in the general, non-clinical population.

The lack of significant left–right differences within each condition could hint that the noted distinction in hemisphere activation reflects state-dependent enhancements in frontal engagement during deceptive behavior rather than stable baseline asymmetries. This pattern could suggest that deceptive responding recruits a distributed frontal control network. Such dynamic, context-sensitive change in hemisphere activation is coherent with contemporary network models of emotion–cognition integration, which emphasize context-dependent recruitment of frontal circuits in response to circumstantial demands rather than fixed structural dominance (Adolphs, 1999; Pessoa, 2017). These results partially support Hypothesis 4, indicating that intentional deceit is associated with increased oscillatory activity coming from the frontal cortex, consistent with enhanced demands on conflict monitoring and inhibitory control during the suppression of truthful outputs and the generation of deceptive alternatives that fit the required circumstances better.

The repeated-measures analysis did not support hemisphere-specific effects or interactions involving hemisphere. Therefore, the present findings should not be interpreted as evidence of frontal lateralization during deceptive responding.

To further evaluate the robustness of the observed associations, additional multiple regression analyses were conducted. Although initial bivariate analyses suggested modest associations between psychological measures and deception-related variables, these relationships were no longer statistically significant after simultaneously accounting for anxiety, depressive symptoms, perceived stress, and LiES scores. Likewise, the regression model predicting relative theta power was not significant, indicating that none of the psychological variables independently explained variability in relative theta activity. Together, these findings suggest that the observed bivariate associations were relatively small and should be interpreted with caution. Given the modest sample size and the exploratory nature of the study, the present results do not support strong conclusions regarding independent psychological predictors of deception-related EEG activity.

Importantly, the increase in right-frontal oscillatory activity remained statistically significant when relative oscillatory power was analyzed. Because relative power normalizes oscillatory activity with respect to total spectral power, this finding indicates that the observed effect cannot be explained solely by global variations in EEG amplitude. Rather, it suggests a selective enhancement of oscillatory dynamics during instructed deception, supporting the robustness of the main electrophysiological finding.

The absence of significant order effects further strengthens the interpretation that increased oscillatory activity reflects the cognitive demands of deceptive responding rather than habituation, fatigue, or practice effects. Regardless of whether participants lied first or told the truth first, deception elicited comparable electrophysiological responses.

The regression analysis indicated that individual differences in anxiety, depression, perceived stress, and the propensity to deceive did not significantly predict relative theta power. Although previous studies have suggested that frontal theta activity may be modulated by emotional or cognitive factors, the present findings do not support an independent contribution of these psychological variables to relative theta power within this sample. The low proportion of explained variance further suggests that variability in relative theta activity is likely influenced by factors beyond the psychological measures included in the model. These findings should be interpreted with caution given the modest sample size and may indicate that relative theta changes observed during deception are more closely related to task-specific cognitive processes than to stable individual differences in affective symptoms or dispositional deception.

Although the repeated-measures ANOVA revealed a significant overall effect of experimental condition, no significant interactions involving hemisphere or frequency band were observed. This pattern suggests that the overall increase in oscillatory activity during deception is relatively generalized across the analyzed bands, although the paired comparisons indicate that the most robust and statistically reliable effect emerged specifically within the right-frontal theta band.

Because the present study employed an instructed laboratory paradigm rather than spontaneous deception, future studies should examine whether similar theta dynamics are also observed during ecologically valid forms of deceptive behavior.

Finally, affective variables and habitual deceptive tendencies did not significantly predict relative theta activity during deception. This suggests that the electrophysiological response associated with instructed deception may primarily reflect transient task-related cognitive mechanisms rather than stable individual psychological characteristics.

## 5. Limitations

Several limitations of the current study should be noted. First of all, the EEG recordings were obtained using a low-density wearable system, which, while presenting advantages in ecological validity and ease of use, provides a more limited spatial resolution and allows less precise source localization within specific frontal subregions. Consequently, the current findings regarding hemispheric and frequency-band effects are less definitively and directly attributed to distinct cortical generators within dorsolateral, ventromedial, or orbitofrontal areas. The use of Muse Headband 2, a low-density consumer EEG device, although it has previous validation studies which have demonstrated acceptable resolution and electrode coverage, precludes precise localization of cortical generators. Therefore, our findings should be interpreted as reflecting frontal scalp oscillatory activity more so than specific frontal cortical mechanisms. Additionally, because participants responded verbally (“yes”/“no”), contamination from facial muscles, jaw activity and speech-related EMG cannot be completely excluded, although the instructions mentioned specifically being as still as possible for the duration of the experiment; however, speech-related muscle activity, movement, response timing, and signal nonstationarity remain alternative explanations.

Secondly, the deception framework employed emotionally neutral and low-stakes inquiries. Experimental control specific to experiments may limit the transfer of results to real-life situations, where deception can often occur in more complex, emotionally charged social contexts or with significant social consequences. Psychophysiological responses may differ in configuration or magnitude under conditions of elevated affective control or motivational importance. Additionally, because the questionnaires are self-reported measures, responses may be influenced by social desirability, recall bias and individual insight, especially when talking about the deception tasks.

Thirdly, the cross-sectional aspects of the questionnaire data prevent causal inferences regarding the relationship between anxiety and habitual deception. While a significant association was observed, it remains unclear whether elevated anxiety increases the tendency to engage in deception, whether frequent deceptive behavior contributes to heightened anxiety, or whether both are controlled by a third variable, such as social sensitivity or trait-level cognitive control.

Fourthly, the sample consisted of healthy young participants, which tends to limit the generalizability of the results to other specific target or age groups. Developmental factors, cultural background, and the presence of affective or personality conditions may change both the psychobiological correlates of deception and the associated frontal interactions. Because of that, it is rather difficult for the results to be generalized to forensic lie detection, clinical populations or high-stakes deception.

Finally, although paired-samples analyses revealed significant activity enhancement in right-hemispheric theta, the effect sizes were in the small-to-moderate range, and hemispheric differences within conditions did not reach statistical significance, in addition to the fact that multivariate analysis did not further support the specific findings regarding bandwidth or lateralization. Replication in larger samples and with higher-density electrophysiological recordings will be suggested in order to confirm the strength and functional specificity of the observed oscillatory model.

## 6. Conclusions

The current study provides psychometric and electrophysiological evidence suggesting that instructed deceptive responding is associated with both affective traits and changes in frontal EEG activity. At the behavioral level, anxiety appeared as a correlate of day-to-day deceptive tendencies, whereas depression and perceived stress did not, suggesting that instructed deception is more closely related to threat sensitivity and anticipatory processes than to general emotionally distressing states. At the electrophysiological level, instructed deceptive responding was associated with increased activity recorded over frontal scalp electrodes. These changes are consistent with functional interpretations previously linking frontal activity to cognitive control, conflict monitoring and inhibitory processing, although the present study cannot directly establish these underlying mechanisms.

Together, the findings suggest that instructed deceptive responding under laboratory conditions is accompanied by frontal EEG changes that are consistent with processes previously associated with executive control. The coupling between anxiety-related emotions and frontal oscillatory patterns may suggest that individual differences in affective regulation shape both the tendency to initiate deceptive behaviors and the electrophysiological responses observed. By suggesting that our findings are consistent with instructed deception being accompanied by coordinated patterns of activity in frontal regions, the current study contributes to the growing literature describing electrophysiological correlates of instructed deceptive responding. Although exploratory, these findings are consistent with models proposing interactions between affective regulation and cognitive control during instructed deceptive responding. The persistence of this effect using relative power provides additional support for the robustness of the primary electrophysiological observation.

These findings should be interpreted as exploratory because of the low-density EEG system, the neutral low-stakes task and the young healthy sample. Furthermore, the use of overt verbal responses may have introduced movement-related artifacts despite applied quality-control procedures. Future studies using high-density EEG and ecologically valid deception paradigms are needed to determine whether these effects generalize to real-world deceptive behavior.

## Figures and Tables

**Table 1:** Descriptive and distributional statistics for demographic variables and psychometric measures (BDI, BAI, PSS, LiES) in the full sample (N = 86).

LiES	Stress	BAI	BDI	Age	
86	86	86	86	86	Valid (N)
0	0	0	0	0	Missing
64.80	35.07	14.05	13.43	26.97	Mean
28.03	12.10	11.36	9.617	9.416	Std. Deviation
0.043	0.663	1.121	0.982	2.193	Skewness
0.260	0.260	0.260	0.260	0.260	Std. Error of Skewness
−1.053	0.184	1.419	0.463	4.853	Kurtosis
0.514	0.514	0.514	0.514	0.514	Std. Error of Kurtosis
0.968	0.957	0.905	0.915	0.737	Shapiro–Wilk
*0.032*	*0.006*	*<0.001*	*<0.001*	*<0.001*	*p*-value of Shapiro–Wilk
14.00	7.000	0.000	1.000	18.00	Minimum
122.0	68.00	56.00	44.00	64.00	Maximum

**Table 2:** Pearson correlation coefficients among psychometric measures (BDI, BAI, PSS, LiES) in the full sample (N = 86). Asterisks represent levels of statistical significance (* *p* < 0.05, *** *p* < 0.001).

*p* FDR	SE Effect Size	Effect Size (Fisher’s z)	Upper 95% CI	Lower 95% CI	*p*	Pearson’s r	
<0.002	0.110	0.884	0.800	0.585	*<0.001*	0.709 ***	BDI vs. BAI
<0.002	0.110	0.791	0.764	0.520	*<0.001*	0.659 ***	BDI vs. PSS
0.264	0.110	0.134	0.336	−0.081	*0.220*	0.134	BDI vs. LiES
<0.002	0.110	0.608	0.677	0.373	*<0.001*	0.542 ***	BAI vs. PSS
0.045	0.110	0.239	0.425	0.024	*0.030*	0.235 *	BAI vs. LiES
0.505	0.110	0.073	0.280	−0.141	*0.505*	0.073	Stress vs. LiES

**Table 3:** Independent-samples *t*-test findings for gender variations among psychometric measures (BDI, BAI, PSS, LiES), including test statistics and effect sizes.

SE Cohen’s d	Cohen’s d	*p*	Df	*t*	
0.263	0.427	*0.104*	84	1.643	BDI
0.265	0.590	*0.026 ^a^*	84	2.271	BAI
0.262	0.361	*0.169*	84	1.389	Stress
0.260	−0.143	*0.583*	84	−0.552	LiES

Note: Student’s *t*-test. ^a^ The Brown–Forsythe test was significant (*p* < 0.05), suggesting a violation of the equal variance assumption.

**Table 4:** Summary statistics of frontal EEG absolute spectral power (μV^2^) across delta, theta, alpha, and beta bands for left (L) and right (R) hemispheres during truthful (Condition 1) and deceptive responding (Condition 2) (N = 48). The table reports central tendency, dispersion, and relative variability indices for each band and condition.

Coefficient of Variation	SE	SD	Mean	Band	Hemisphere
0.168	1.895	13.132	77.96	alpha1	Left (L)
0.163	1.891	13.102	80.37	alpha2	
0.216	2.529	17.524	81.29	beta1	
0.204	2.442	16.921	83.03	beta2	
0.183	2.165	15.000	81.88	delta1	
0.178	2.167	15.013	84.54	delta2	
0.179	1.893	13.113	73.22	theta1	
0.210	2.271	15.731	74.86	theta2	
0.154	1.825	12.645	82.27	delta1	Right (R)
0.135	1.641	11.366	84.49	delta2	
0.145	1.635	11.326	78.20	alpha1	
0.120	1.393	9.652	80.71	alpha2	
0.177	2.060	14.272	80.55	beta1	
0.170	2.003	13.876	81.67	beta2	
0.174	1.800	12.474	71.88	theta1	
0.171	1.881	13.032	76.15	theta2	

**Table 5:** Comparisons of frontal EEG absolute spectral power between truthful (Condition 1) and deceptive responding (Condition 2) for left (L) and right (R) hemispheres across alpha, beta, delta and theta frequency bands (N = 48).

*p* FDR	SE Cohen’s d	Cohen’s d	*p*	df	*t*	Frequency Band (Condition 1 vs. Condition 2)	Hemisphere
0.258	0.105	−0.257	*0.081*	47	−1.781	alpha	Left (L)
0.258	0.064	−0.231	*0.117*	47	−1.597	beta	
0.258	0.120	−0.216	*0.142*	47	−1.494	delta	
0.397	0.120	−0.134	*0.357*	47	−0.930	theta	
0.245	0.119	−0.292	*0.049*	47	−2.020	alpha	Right (R)
0.348	0.073	−0.158	*0.278*	47	−1.097	beta	
0.258	0.128	−0.209	*0.155*	47	−1.445	delta	
0.040	0.116	−0.436	*0.004*	47	−3.022	theta	

Note: Student’s *t*-test.

**Table 6:** Analysis between left (L) and right (R) frontal EEG absolute spectral power during deceptive outputs (Condition 2) across alpha, beta, delta, and theta frequency bands (N = 48). Test statistics and significance levels for hemispheric differences are reported for each frequency band.

*p*	df	*t*	Frequency Band (Left vs. Right Hemisphere)
*0.863*	47	−0.174	Alpha
*0.525*	47	0.640	Beta
*0.982*	47	0.023	Delta
*0.560*	47	−0.587	Theta

Note: Student’s *t*-test.

**Table 7:** Comparison between left (L) and right (R) frontal EEG absolute spectral power during truthful responding (Condition 1) across alpha, beta, delta, and theta frequency bands (N = 48). Test statistics and significance levels are reported for each frequency band.

*p*	df	*t*	Frequency Band (Left vs. Right Hemisphere)
0.916	47	−0.106	Alpha
*0.730*	47	−0.347	Beta
*0.868*	47	−0.167	Delta
*0.523*	47	−0.644	Theta

Note: Student’s *t*-test.

**Table 8:** Relative theta power during truthful and deceptive responding.

*EEG Measure*	Truth Mean & SD	Deception Mean & SD	t(47)	*p*	Cohen’s d
Relative Theta	0.19, 0.02	0.19, 0.02	−2.76	0.01	0.40

Note: Student’s *t*-test.

**Table 9:** The 2 (Condition: truthful vs. deceptive) × 2 (Hemisphere: left vs. right) × 4 (Band: delta, theta, alpha, beta) repeated-measures ANOVA. Test statistics and significance levels are reported for each level.

*Effect*	F	*p*	ηp^2^
Condition	5.87	0.02	0.11
Hemisphere	0.01	0.93	0.00
Band	14.72	0.00	0.24
Condition × Hemisphere	0.08	0.78	0.00
Condition × Band	0.88	0.44	0.02
Hemisphere × Band	0.55	0.58	0.01
Condition × Hemisphere × Band	1.98	0.13	0.04

Note: Repeated-measures ANOVA.

**Table 10:** Multiple linear regression predicting LiES. Model statistics: R^2^ = 0.11, adjusted R^2^ = 0.05, F(3, 44) = 1.81, *p* = 0.16.

*Predictor*	β	*t*	*p*
BAI	0.01	0.04	0.97
Stress	−0.21	−1.08	0.29
BDI	0.42	1.64	0.11

Note: Multiple linear regression.

**Table 11:** Multiple linear regression predicting relative theta power.

*Predictor*	β	*t*	*p*
BAI	0.14	0.49	0.62
PSS	−0.17	−0.87	0.39
BDI	0.17	1.06	0.30
LiES	−0.14	−0.59	0.56

Note: Multiple linear regression.

## Data Availability

The raw data supporting the conclusions of this article will be made available by the authors, without undue reservation.

## Abbreviations

The following abbreviations are used in this manuscript:

BAI	Beck Anxiety Inventory
BDI	Beck Depression Inventory
CI	Confidence interval
CQT	Control Question Test
CV	Coefficient of variation
EEG	Electroencephalography
FDR	False Discovery Rate
FFT	Fast Fourier Transform
JASP	Jeffreys’s Amazing Statistics Program
LiES	Lying in Everyday Situations Scale
PSS	Perceived Stress Scale
